# Psychological Adaptations to High-Intensity Interval Training in Overweight and Obese Adults: A Topical Review

**DOI:** 10.3390/sports10050064

**Published:** 2022-04-22

**Authors:** Alexios Batrakoulis, Ioannis G. Fatouros

**Affiliations:** Department of Physical Education and Sport Science, University of Thessaly, 42100 Trikala, Greece; ifatouros@uth.gr

**Keywords:** intermittent exercise, obesity, psychological responses, mental health, adherence, enjoyment, affect, quality of life, anxiety, depression

## Abstract

Regular exercise has been reported as a fundamental piece of the management and treatment puzzle of obesity, playing a vital role in numerous psychological indicators. However, it is unclear whether high-intensity interval training (HIIT) can improve critical psychological health markers such as adherence, exercise enjoyment, affective responses, health-related quality of life, anxiety, and depression in overweight and obese adults. The purpose of this topical review was to catalogue studies investigating the psychological responses to HIIT in order to identify what psychological outcomes have been assessed, the research methods used, and the results. The inclusion/exclusion criteria were met by 25 published articles investigating either a traditional, single-component (84%) or a hybrid-type, multi-component (16%) HIIT protocol and involving 930 participants with overweight/obesity. The present topical review on HIIT-induced psychological adaptations shows that this popular exercise mode, but also demanding for the masses, can meaningfully increase the vast majority of the selected mental health-related indices. These improvements seem to be equal if not greater than those observed for moderate-intensity continuous training in overweight and obese adults. However, further research is needed in this area, focusing on the potential mechanisms behind positive alterations in various psychological health parameters through larger samples and high-quality randomized controlled trials.

## 1. Introduction

### 1.1. Obesity: A Public Health Challenge

Obesity is a multifactorial metabolic disease inducing adverse alterations in adipose tissue and predisposing metabolic dysregulation, which is responsible for an increased risk of other lifestyle-related chronic diseases [1]. More than one in four adults have obesity, almost two in three adults are overweight worldwide [2], and the vast majority of them are middle-aged and older adults [3]. It is noteworthy that the annual global medical cost of treating obesity-related illness has been projected to be US $2.0 trillion, creating a global concern and insecurity [4]. The pathogenesis of obesity is multifactorial and is characterized by an abnormal level of body weight and adiposity. Such a condition may be sufficiently excessive to damage health while resulting in numerous health complications, affecting various psychological indices [5]. Interestingly, overweight/obese adults are likely to avoid regular exercise in a gym setting due to weight bias, which is linked to insufficient physical activity levels [6], poor mental health [7], impaired motivation, and exercise-related perceived competence [8]. Depression and anxiety are also important factors that limit these populations in participating in an exercise program, underlining the psychosocial burden of obesity [9,10]. Weight maintenance also appears to be a challenging, long-term goal highly affected by various psychological health factors [11]. However, a tough question regarding the feelings of exercisers is still unanswered, since the vast majority of the adult population are insufficiently active [12].

### 1.2. Exercise, Obesity and Mental Health

The triangle of obesity, inactivity, and poor psychological health commonly observed in adults may be a critical issue that may lead to impaired mental health [13,14]. Although exercise alone does not seem to significantly improve symptoms of mental health or body weight in adults with serious mental illness [13,15,16,17], it has been documented as a valuable piece of the treatment puzzle in populations with overweight/obesity and impaired mental health [13,14]. However, only one in three adults are classified as sufficiently physically active worldwide [18], and thus physical inactivity has been reported as one of the greatest threats to global public health and is linked to the most prevalent lifestyle-related chronic diseases [19]. As such, various structured exercise modes for weight management and health promotion are currently underlined as some of the most popular trends for exercise professionals and their clients in the health and fitness industry worldwide [20]. Given that inactivity is correlated with depression [21] and obesity [6], there is strong evidence supporting the vital role of regular exercise in this population. Specifically, the current exercise prescription guidelines for this population highlight the importance of incorporating both moderate-intensity continuous training (MICT) (at least 300 min per week) and resistance training (RT) (at least two whole-body sessions per week) into a single session or as separate weekly sessions [22]. Both training modalities have been reported feasible, safe, and effective for overweight/obese individuals; however, compliance rates are low when this population engages in such traditional exercise interventions without supervision in a real-world gym setting [23]. Given that lack of time has been identified as the primary exercise barrier in adults [24,25,26], time-consuming exercise approaches such as MICT and RT appear less attractive to individuals of an unhealthy weight [27].

### 1.3. High-Intensity Interval Training and Obesity

**Figure 1:**
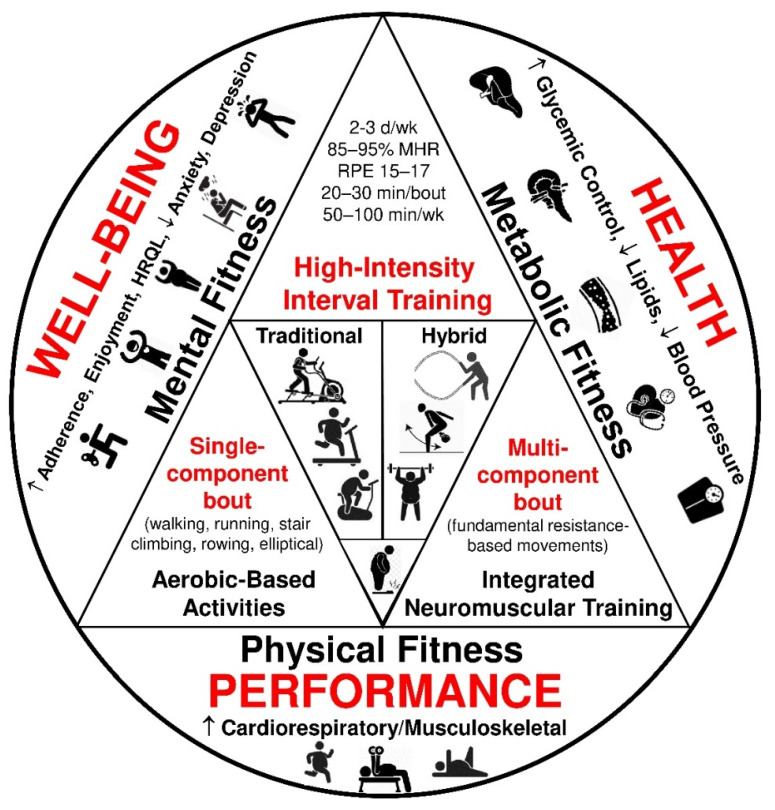
The effects of high-intensity interval training on health, performance, and well-being. MHR: maximum heart rate; RPE: rating of perceived exertion; HRQL: health-related quality of life. [13,29,30,31].

High-intensity interval training (HIIT) for clinical populations, including people experiencing obesity, is generally defined as a cardiovascular exercise strategy composed of repeated bouts of brief, intermittent, intense exercise [85–95% of heart rate reserve (HRR); rating of perceived exertion: 15–17] followed by periods of passive or active recovery (60–70% HRR) [28]. In general, there are two mainly used HIIT program design formats implemented for populations with obesity: (1) traditional, aerobic-based training as a single-component session and (2) hybrid, resistance-based training as a multi-component session (Table 1) [29,30,31,32]. Both HIIT models are feasible, effective, and popular exercise approaches for the masses in the fitness community [33]. According to the American College of Sports Medicine, the role of HIIT in cardiometabolic disease prevention is vital since HIIT shows similar improvements in body composition, glycemic control, blood lipid profile, and blood pressure as MICT in overweight and obese adults [34]. However, emerging evidence suggests that such exercise modes may be demanding for previously inactive individuals with unhealthy weight [35]. Thus, HIIT-based protocols should be adjusted to exercisers’ psychological and physiological profiles aiming to be widely adopted by the masses in a free-living environment [36]. Figure 1 summarizes the effects of HIIT on metabolic health, physical performance, and well-being in overweight and obese adults [13,29,30,31].

The objective of the present topical review was to summarize the research methods used and the results reported in studies where selected psychological outcomes (e.g., adherence, affect valence, exercise enjoyment, depression, and anxiety) were examined. Such a review article may disseminate the main research findings on psychological responses to HIIT in a critical population such as the overweight and obese adults, aiming to identify research issues, considerations, and gaps in the literature. With research on psychological responses to HIIT being scarce, all published research on the topic was captured, regardless of the study design, aiming to provide a comprehensive perspective on the existing evidence. The topical review addressed the general question: ‘What is known from published research about selected psychological adaptations to various forms of HIIT in overweight and obese adults?’.

## 2. Methods

### 2.1. Literature Search Strategy

Articles were retrieved from PubMed/MEDLINE from inception up to 15 February 2022 after a systematic electronic search by the two authors (A.B. and I.G.F.). Examples of the search terms included: overweight, obese, obesity, interval or intermittent training or exercise, high-intensity interval training or exercise, HIIT, HIIE, mental health, perceptual, psychological, arousal, feeling, depression, anxiety, mood, quality of life, affect, enjoyment, compliance, adherence. The complete search strategy is available in the online supplemental material (Table S1). Reference lists from articles and other related resources were scanned for any additional relevant articles.

### 2.2. Eligibility Criteria

Studies were considered eligible for inclusion if the following criteria were met: (1) participants were adults aged 18–64 years, with no diagnosed comorbidities or signs/symptoms of any non-communicable disease, and with a BMI ≥ 25 kg/m^2^; (2) included studies employed an intervention of various forms of HIIT; and (3) examined at least one of the following psychological outcomes in humans: adherence, affect valence, exercise enjoyment, health-related quality of life (HRQL), depression, and anxiety. All studies were required to be written in English, and published in a refereed journal from inception up to 15 February 2022. Considering the exclude criteria, the following were excluded: (1) studies involving a mixed sample of individuals with overweight/obesity and other non-communicable diseases per intervention arm; (2) studies involving children/adolescents (<18 years old) or older adults (>64 years old); (3) articles where the effects of HIIT intervention cannot be isolated because HIIT was involved as part of a multi-component intervention (e.g., a training program consisting of HIIT and MICT or resistance training or a diet or psychological intervention); (4) articles that did not assess the outcome measures of interest; (5) any non-human studies; (6) studies published in languages other than English; and (7) studies that had not undergone full peer review (e.g., conference proceedings, posters, published abstracts, lay articles, proposed studies, dissertations, theses, reviews, commentaries, and debates).

### 2.3. Study Selection

**Figure 2:**
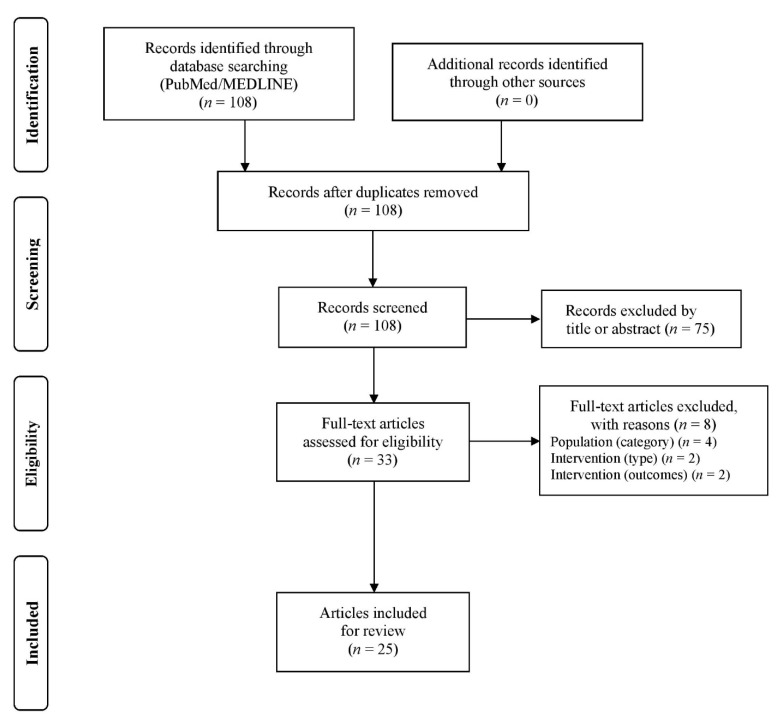
Flowchart of the systematic literature search.

The two authors (A.B. and I.G.F.) independently screened the titles and abstracts of potentially eligible studies and downloaded the full texts of the remaining articles to assess their eligibility. Any discrepancies between the two authors were resolved by discussion and consultation with a research fellow (A.Z.J.). EndNote X9 (Clarivate Analytics, Philadelphia, PA, USA) literature management software was used to manage the literature search records. The flow diagram is illustrated in Figure 2, showing the literature search and selection process in detail.

### 2.4. Data Extraction

The two authors (A.B. and I.G.F.) independently extracted data using Microsoft Excel. Any disagreements were resolved by consensus. In case of insufficient information, the authors of the included studies were contacted via email for missing values where required. Data extraction included first author, year of publication, country, intervention duration, sample size, participant demographics (e.g., gender, mean age, and activity level), study design, HIIT classification (traditional or hybrid), HIIT intervention details (frequency, intensity, time, and type), and critical psychological outcome measures and findings reported from each eligible study, as shown in Table 2. 

## 3. Results

### 3.1. Articles Retrieved

The electronic search yielded 108 articles. After screening of titles, abstracts, and full texts, 25 eligible studies were included in this review (Figure 2). Pertinent data extracted from each article are presented in Table 2. 

### 3.2. Article Characteristics

Articles were published from 2010 to 2022 and research was conducted in 11 countries. There was a total sample of 930 sedentary/inactive participants with overweight/obesity across all studies. Twenty-two (88%) articles reported on the investigation of psychological measures as a primary outcome, while in three (12%) articles, the psychological measures were a secondary outcome. Six (24%) articles reported on investigations of acute responses to HIIT protocols, while nineteen (76%) articles reported on the effects of chronic responses (≥2 weeks) of HIIT. Training studies lasted from 2 to 48 weeks in duration, with exercise session frequency ranging from three to five times per week, and used quantitative methods. Articles reported on studies that implemented between-subject designs (*n* = 11, 44%), within-subject designs (*n* = 8, 32%), randomized controlled trials (*n* = 5, 20%), or a comparative study design (*n* = 1, 4%). Articles reported on studies that assigned supervised (*n* = 21, 84%), semi-supervised (*n* = 3, 12%), or unsupervised (*n* = 1, 4%) HIIT interventions. Articles reported on studies that were conducted in a lab-based (*n* = 16, 64%), semi-field (*n* = 4, 16%), or field-based (*n* = 5, 20%) environment.

### 3.3. Exercise Protocols

Of the 25 HIIT protocols applied in the reviewed studies, 21 (84%) were classified as traditional (single-component) and four (16%) were classified as hybrid (multi-component). HIIT protocols did not demonstrate a considerable variation in duration, intensity, and rest period. The most frequently reported HIIT protocol was a traditional protocol consisting of 10 × 30–90 s high-intensity work intervals interspersed by 30–120 s active recovery periods (*n* = 14). Regarding exercise modality, 14 studies (56%) conducted HIIT protocols using cycle ergometers, 7 studies (28%) used treadmills, and 4 studies (16%) utilized body-weight resistance.

## 4. Psychological Adaptations

### 4.1. Adherence

Several exercise programs with different training parameters have been suggested to enhance long-term adherence in overweight/obese individuals aiming to promote an active lifestyle through a permanent behavior modification [37]. Adherence is linked to affective responses to exercise intensity since enjoyment decreases with elevating intensity. Such an observation may be supported by the fact that the vast majority of adults worldwide do not meet the physical activity criteria [38]. The exercise experience utilizing HIIT regimens for individuals with obesity remains largely undecided. Current evidence suggests numerous exercise training strategies for promoting long-term adherence to weight management programs among overweight and obese individuals. Consistency is a critical factor for these populations seeking to accomplish health-related lifestyle and behavioral changes, and thus latest international physical activity and sedentary behavior guidelines highlight the vital role of an active lifestyle [37]. However, most adults with an unhealthy body mass demonstrate lower exercise adherence and higher dropout rates than normal weight individuals to these programs [39].

For HIIT, evidence exists that both supervised [23,40] and unsupervised programs can achieve high adherence rates in middle-aged overweight and obese men [41]. Moreover, individuals with abdominal obesity exhibited significantly higher adherence to a 4-week HIIT program compared with MICT, showing that HIIT may be a feasible exercise strategy for populations not only at risk of metabolic diseases [27] but also for those impacted by obesity alone [42,43]. It is also notable that no differences were found between HIIT and MICT concerning their influence on adherence in adults with unhealthy weight following an 8-week intervention [44,45]. HIIT has also been reported as a well-tolerated and accepted training modality for previously inactive overweight and obese adults [40,46]. HIIT-based regimens were associated with both marked increases in adherence rates and with meaningful improvements in exercise behavioral regulation in overweight/obese women following a 5-, 10-, and 12-month supervised, hybrid-type HIIT intervention [40,47,48,49,50]. In summary, an adherence rate of ≥80% was reported for both HIIT and MICT in 13 studies investigating the effects on body composition in overweight/obese individuals [51]. However, it is not clear if HIIT is highly associated with high compliance and low dropout rates in adults with obesity since the literature presents conflicting evidence [52]. In particular, higher exercise volume is linked to higher dropout rates in HIIT interventions [53]. Thus, low-volume HIIT regimens may be a valuable solution to overcome time commitment-related barriers to regular exercise in the real world.

### 4.2. Affective Responses

Affect is an instinctive mood response caused without considerable thought and is associated with pleasure or displeasure and tension or calmness [54]. It is still unclear if HIIT is superior to MICT in inducing more favorable affective responses in people with obesity. The feasibility of traditional HIIT-type protocols in previously inactive overweight/obese adults has been questioned due to their inherent intense nature showing different affective responses compared to normal weight individuals and MICT [55,56]. Low exercise volumes are associated with better feelings of pleasure in HIIT protocols, but they declined in individuals without exercise experience [57,58]. Current evidence shows that HIIT does elicit similar affective levels compared to MICT [41,45,59,60,61,62] and much higher levels compared to high-intensity continuous training in populations with unhealthy body mass [63]. It is noteworthy that shorter interval bouts (≤30 s), despite the supramaximal intensity, were associated with less aversive affective valence than more prolonged bouts (60–120 s) characterized by the lower intensity in inactive overweight and obese adults [63,64]. Such a psychological attribute may underline the positive role of HIIT in offering a less monotonous and more engaging exercise experience compared with MICT.

### 4.3. Exercise Enjoyment

Enjoyment is a psychological state that is not elicited reflexively or instinctively, but after appraising or cognitively evaluating a situation [54]. There is no robust evidence showing that HIIT is more effective than MICT for increasing enjoyment in adults with obesity. Generally, middle-aged obese women showed lower pleasure rates than normal weight and overweight women during an incremental exercise test [55]. It is also widely accepted that feelings of pleasure and enjoyment are crucial factors for adherence to exercise programs [38]. Thus, insufficiently active overweight/obese individuals having pleasant exercise experiences, demonstrated significantly lower dropout and higher compliance rates than those individuals who engaged in unpleasant exercise regimens [63]. Noticeably, HIIT has been documented as an aversive and not widely feasible exercise type among inactive populations with overweight and obesity, showing lower pleasure and enjoyment than traditional MICT [65,66]. In contrast, both single- [67,68] and multi-component [44] HIIT regimens appear to be more effective in increasing the intention to implement such programs in the future compared to combined aerobic and resistance training, even without elevating individual enjoyment levels. Of particular importance is that HIIT protocols with short work intervals (≤30 s) induced greater enjoyment than those characterized by long work intervals (60–120 s) in untrained overweight and obese individuals [63,64]. This observation may highlight the beneficial effect of HIIT on a pleasant workout compared with MICT, which is more common among normal weight individuals [69,70,71]. However, single-component HIIT protocols seem to be similarly effective to MICT at improving exercise enjoyment [60,61,62].

### 4.4. Health-Related Quality of Life

Obesity is correlated with declined levels of HRQL [72]. Notably, sufficiently active individuals with obesity demonstrate higher HRQL than those classified as sedentary, despite the similar body mass status [73]. The grade of obesity negatively influences an individual’s HRQL, showing that higher obesity levels are associated with lower HRQL levels [74]. Further, poor HRQL levels are associated with impaired physical health, but not necessarily related to lowered mental well-being levels in persons with overweight/obesity [75]. Limited data are available regarding the efficacy of HIIT on HRQL in populations with obesity. However, studies investigating the impact of low-volume HIIT protocols on specific dimensions of HRQL (e.g., vitality, social functioning, and mental health) indicated meaningful improvements in overweight and obese individuals following either short- or long-term interventions [40,43,46,76,77,78,79,80]. Similarly, HIIT demonstrates beneficial effects on HRQL in other populations such as healthy inactive adults [81,82] and sedentary people with type 1 diabetes mellitus [83] or coronary artery disease [84], but these adaptations are not different from those observed for MICT [78]. It is important to note that people achieving the optional physical activity levels are likely to have higher HRQL levels compared to inactive individuals [85,86]. In studies investigating HIIT, despite the low weekly training time commitment (50–100 min per week) [29] compared to current guidelines on physical activity and sedentary behavior (150–300 min per week) [87], HIIT appears to elevate both physical and mental health components. Thus, the positive psychological effects of HIIT on inactive populations also highlight an impactful increase of HRQL levels in those engaging in both short- and long-term HIIT protocols.

### 4.5. Anxiety

Obesity has been documented as a risk factor for anxiety disorders due to weight-related discrimination and psychological distress in obese individuals [16]. Additionally, people with unhealthy weight demonstrate poor levels of physical functioning ability, negatively affecting activities of daily living and quality of life. As such, living with obesity seems to be not only a high-risk condition for developing several cardiometabolic health abnormalities [5], but also a stressful condition for this population [88]. There is little evidence for dose–response effects of physical activity on anxiety and depression, showing that training parameters such as frequency and duration cannot change the adaptations observed from various aerobic and resistance training protocols [89]. Regular exercise may be a helpful tool for lowering anxiety levels in several populations, including the obese [13]. However, there is scarce research on the effects of exercise on anxiety in adults with obesity. Hence, the existing data are limited while reporting inconsistent findings. Thus, drawing robust conclusions regarding this psychological outcome remains to be questioned. Future research is warranted to explore further the effectiveness of HIIT-based interventions in this cohort, aiming to reveal evidence that could be interpreted into real-world conditions. Additionally, more studies are needed in this area, investigating HIIT-induced changes in anxiety, focusing on populations with unhealthy weight and anxiety above the normal levels [13].

### 4.6. Depression

Depression is a severe mental health disorder more commonly observed in sedentary individuals as well as obese adults compared to physically active individuals and normal weight adults [21]. The link between obesity and depression has been well studied, showing that poor self-image, low self-esteem, ostracism, and discrimination may be the principal factors increasing depression levels in this population [90]. Weight bias and stigma are also critical reasons enhancing the evidence that individuals with obesity are likely to demonstrate depressive disorders compared to those with healthy weight [10]. Physical activity and exercise appear to reduce depression symptoms comparable to those of antidepressants in individuals with acute or chronic depression [91]. Interestingly, both depressed and non-depressed patients with obesity demonstrate similar physical activity levels, highlighting the crucial role of obesity in mental health and particularly in depressive disorders [92]. However, exercise alone does not induce beneficial alterations in depression in adults with obesity [13]. Such a finding may be supported by studies investigating the exercise effects on depression in overweight/obese individuals with no abnormal depression levels at baseline [79]. No meaningful improvements were observed in depression for those subjects following any exercise intervention, including HIIT [13]. Nevertheless, there is evidence that HIIT may play a positive role in improving depression symptoms in populations with mood barriers [13,15,93]. Such an outcome may be partly explained by the crucial role of endocrine responses related to enjoyment and pleasure feelings supported by serotonin secretion and an increase of brain-derived neurotrophic factors [94]. It is noteworthy that an 8-week HIIT intervention (thrice per week) elicited a significant reduction in the score of negative moods, tension, and depression in overweight/obese young men [93]. On the other hand, an 8-week HIIT program resulted in a meaningful decrease in depressive symptoms but not anxiety levels in healthy and physically active young adult women [95].

## 5. Discussion

Through a topical review, 25 articles were systematically identified that reported psychological outcomes associated with HIIT in overweight/obese adults. The vast majority of eligible studies included in this review investigated various forms of HIIT in a supervised (84%) and lab-based setting (64%). The finding that 68% (17/25) of the eligible articles were published between 2016 and 2022, while 11 studies (44%) were published in 2019–2022, shows that this topic may be a rapidly emerging area of health psychology research. This review summarized and synthesized the existing literature to facilitate further discussion of the issues, considerations, and gaps that need to be addressed in future research attempts. Specifically, the selected psychological measures were synthesized into six categories: adherence, affective responses’ valence, exercise enjoyment, health-related quality of life, anxiety, and depression.

Overall, affect and enjoyment have been the most frequently studied psychological outcomes in the HIIT literature for overweight/obese individuals. The main results in the present topical review show that participants experienced equal or greater affect and enjoyment of HIIT in comparison to MICT. This is an important finding underlining the preference of more vigorous, but less time-consuming, exercise protocols. As such, the exercise volume appears to be a critical training parameter since it affects the feeling of pleasure for HIIT and MICT. This observation is also supported by the fact that the number of work bouts may play some role in whether a HIIT protocol would be pleasant or not [58]. Interestingly, HIIT studies examining the chronic effects on affect and enjoyment in overweight and obese populations mainly reported positive changes [44,60,67,68]. This finding is not aligned with the outcomes reported by the studies that investigated the acute effects on those psychological markers in this cohort [55,65]. Taking this observation into account, it seems that familiarization and progressive overloading may be a critical role in the feeling of pleasure to HIIT among previously inactive adults of unhealthy weight.

Considering that mental health disorders are common among overweight and obese adults [15,16,17,96], an in-depth investigation of the efficacy of exercise training on numerous psychological health markers may be critical for evaluating the role of regular exercise in the management and treatment of obesity [13,15]. Recent data show that exercise leads to beneficial changes in various psychological outcome measures such as quality of life, vitality, and mental health [13,15]. Nevertheless, no meaningful differences were found in depression, anxiety, and perceived stress in populations with overweight and obesity following various exercise interventions [13]. In general, the effectiveness of exercise training on psychological health in overweight/obese individuals has been poorly studied, and there is no strong evidence regarding the comparative effectiveness of different types of exercise in this area [97,98]. However, exercise types, as well as gender, appear to moderate psychological effects in this cohort, due to the large variability in intervention characteristics [13].

It is not clear in the literature whether HIIT-based programs are effective for long-term motivation, behavioral modification, vitality, enjoyment, and adherence to exercise participation in previously inactive overweight and obese adults in a real-world gym setting [46]. On the one hand, HIIT appears to lower anxiety and depression in populations suffering from physical and mental health issues [99]. On the other hand, resistance training has been documented as an effective exercise type for improving various psychological outcome measures in populations with overweight and obesity. These improvements appear comparable and sometimes more significant than those observed for MICT interventions [98]. It is noteworthy that according to the international guidelines on physical activity and exercise, a multi-component approach integrating aerobic and muscle-strengthening activities either in a single session or in the same week as two different types of stimuli has been suggested for overweight and obese individuals [22,87,100]. Nevertheless, such an exercise approach appears to be time-consuming and less motivating, and thus low adherence and high attrition rates have been reported for populations with unhealthy weight when engaging in long-term exercise interventions [101]. Considering that lack of time has been reported as the primary perceived exercise barrier [24,26], while the low adherence rates to long-term engagement in prescribed physical activity is common among inactive individuals with unhealthy weight [102], it seems that there is a place for a time-efficient training modality such as HIIT in the exercise programming puzzle for overweight and obese individuals. Such an exercise strategy may be a viable alternative to MICT [103,104], after addressing special considerations and safety issues related to the implementation of HIIT for this particular population.

### 5.1. Future Research

The small number of available investigations provides limited data regarding the effectiveness of HIIT on selected mental health-related outcome measures in adults with overweight/obesity. Overall, the viability of HΙΙΤ as a widely used exercise strategy for populations with obesity remains arguable. More rigorous research is required to identify whether HIIT can play some role as part of the solution to the global problem of obesity. However, from a physiological standpoint, the current evidence supports the viability of HIIT as an alternative to MICT, since both exercise modalities demonstrate similar benefits in various psychological health markers in this cohort [103]. Further research is warranted in this area, focusing on investigating the dose–response relationship of long-term HIIT interventions, and mental health status for individuals with obesity in a free-living environment, as previously reported [105]. In addition, the potential mechanisms behind positive alterations in various psychological health parameters should be investigated in the future through larger samples and high-quality randomized controlled trials conducted in a real-world environment.

Considering that data regarding the effectiveness of HIIT on anxiety, depression, and mood state are currently limited for this population, the findings briefly summarized here may lead researchers to study HIIT-based interventions promoting positive alterations in selected mental health indicators in insufficiently active adults with unhealthy weight. Such a strategy may be a valuable path to discover how to engage the masses in effective and injury-free regimens characterized by a serious lack of time commitment. Lastly, additional randomized controlled trials with a focus on compliance and adherence to such a vigorous and challenging type of exercise are needed, given that HIIT [66], as well as sprint interval training [106], have been highlighted as somewhat inappropriate for a largely sedentary population. With its recent rise in popularity around the globe [20,33] and concerns of whether HIIT can be adhered to long-term [37,55], the feasibility of this exercise type remains unclear. Considering that both physiological [28,29,34] and psychological benefits of HIIT in obesity have been extensively reported, the practical use of such a non-traditional exercise modality can only be obtained if individuals with overweight/obesity are sustainably engaging in a HIIT experience in various settings and without supervision.

### 5.2. Strengths and Limitations

On the one hand, this brief review has several strengths, including the use of rigorous, systematic methods for searching, assessing, and synthesizing the research evidence; the classification (traditional or hybrid) of HIIT protocols administered across all studies; the provision of reasonable suggestions for further research in this rapidly emerging area of health psychology research; and the summary as well as dissemination of key research findings regarding the psychological responses to HIIT in a population that currently represents the vast majority of adults in the Western world. On the other hand, the present study has some limitations that should also be acknowledged. Precisely, it does not appraise the quality of evidence in the primary research reports and, thus, this topical review provides a narrative explanation of existing research without addressing the synthesis of available data. A review study of this kind is mainly concentrated on a greater range of study designs and methodologies than a systematic review, which is likely to focus on a detailed analysis of a smaller number of randomized control trials.

## 6. Conclusions

HIIT is a time-efficient strategy to provoke psychological adaptations linked to increased exercise adherence and enjoyment through short- or long-term interventions in adults with overweight/obesity. Importantly, supervision may play a critical role in achieving exceptional high compliance to HIIT programs in real-world settings. Although HIIT has been documented as a demanding exercise mode for the general public, individuals with obesity show beneficial changes in HRQL following progressive HIIT protocols. In summary, current evidence suggests that brief, vigorous, intermittent exercise can be an enjoyable and influential part of the exercise programming puzzle for this population when no comorbidities or additional cardiometabolic health risk factors are existent. However, further randomized controlled trials implementing semi-supervised or unsupervised HIIT interventions conducted in a field-based environment are needed to investigate the real-world effectiveness of HIIT on a broad spectrum of psychological health indicators in overweight and obese adults.

## Figures and Tables

**Table 1:** Classification of common HIIT variations.

Model	Format	Training Parameters	Modalities
Traditional	Single-component (aerobic-based)	Frequency: 2–3 days per week Work intervals: 2–4 min or 30–90 s (85–100% HRmax; RPE 15–17) Recovery intervals: 1–3 min or 30–90 s (60–70% HRmax; RPE 11–13) Series per session: 4–6 or 8–10 times Progression of work-to-rest ratios: 0.75:1 (weeks 1–6), 1:1 (weeks 7–12), and 1:0.75 (weeks 13–16) Total time: 20–30 min	One of the following: walking running cycling stair climbing elliptical rowing swimming
Hybrid	Multi-component (resistance-based)	Frequency: 2–3 days per week Work intervals: 30–60 s (85–100% HRmax; RPE 15–17) Recovery intervals: 30–60 s (passive; RPE 11–13) Series per session: 8–12 times (2–3 rounds) Recovery time per round: 2–3 min Progression of work-to-rest ratios: 1:3 (weeks 1–6), 1:2 (weeks 7–12), and 1:1 (weeks 13–16) Total time: 20–30 min	Full-body movements using either body-weight ^1^ or integrated neuromuscular exercises ^2^ with adjunct equipment such as kettlebells medicine balls suspension exercise devices battle ropes resistance bands balance balls stability balls

HRmax: maximum heart rate; RPE: rating of perceived exertion. ^1^ Low knee skips, hops in place, jogging in place, jumping jacks, split jacks, ice skaters, mountain climbers, and burpees [29]; ^2^ Integrated neuromuscular movements using fundamental patterns (e.g., bend and lift, pushing, pulling, carry, single-leg, and twist).

**Table 2:** Data extracted from each article included for review.

Article	Country	Duration (wks.)	Sample ^1^ N/F/M	Mean Age ± SD (yrs.)	Activity, BMI Classification	Study Design	HIIT Classification	Summary Description of HIIT Intervention (Frequency, Intensity, Time ^2^, Type)	Psychological Findings	Dropout ^3^
Arad (2020)	United States	14	28/28/0	29.0 ± 4.0	Sedentary, Overweight/ Obese	Chronic, RCT	Traditional (single- component)	3 d/wk.; work intervals: 75–90% HRR, 30–60 s; work/recovery ratio: 1:7–1:3; 24 min; cycling (supervised, lab-based)	adherence (↓)	35%
Arboleda-Serna (2022)	Colombia	8	35/35/0	29.6 ± 7.7	Active, Overweight	Chronic, RCT	Traditional (single- component)	3 d/wk.; work intervals: 15 × 30 s (90–95% HRmax), rest intervals: 60 s (50–60% HRmax), 21.5 min, walking, jogging, or running (supervised, field-based)	quality of life (↔)	0%
Astorino (2019)	United States	6	19/19/0	37.0 ± 10.0	Inactive, Obese	Chronic, within- subject	Traditional (single- component)	3 d/wk., work intervals: 6–10 × 60–120 s (70–110% PPO), rest intervals: 5–9 × 60–120 s, 19–26 min, walking, cycling, jogging, rowing, or elliptical machine (supervised, semi-field)	enjoyment (↔), affect (↔)	11%
Batrakoulis (2020)	Greece	40	49/49/0	36.4 ± 4.4	Inactive, Overweight/ Obese	Chronic, RCT	Hybrid (multi- component)	3 d/wk., work intervals: 8–10 × 20–40 s (73–88% HRmax), rest intervals: 20–40 s (passive), 1–3 rounds, 23–41 min, integrated neuromuscular training (supervised, field-based)	adherence (↑), vitality (↑), distress (↓)	11%
Boyd (2013)	Canada	3	19/0/19	22.7 ± 3.9	Sedentary, Overweight/ Obese	Chronic, between-subject	Traditional (single- component)	3 d/wk., work intervals: 8–10 × 60 s (70–100% PWR), rest intervals: 7–9 × 60 s (active/low-intensity), 15–19 min, cycling (supervised, lab-based)	adherence (↑), enjoyment (↑), affect (↑)	0%
Cheema (2015)	Australia	12	12/7/5	39.0 ± 17.0	Inactive, Overweight/ Obese	Chronic, between-subject	Hybrid (multi- component)	4 d/wk., work intervals: 2 min (>75% HRmax), rest intervals: 1 min (standing/pacing), 40 min, boxing (supervised, field-based)	adherence (↑), quality of life (↑)	0%
Chu (2021)	Taiwan	–	60/60/0	22.1 ± 2.0	Inactive, Overweight/ Obese	Acute, within- subject (cross-over)	Traditional (single- component)	1 bout, work intervals: 10 × 45 s (Wmax), rest intervals: 9 × 75 s (50 watts), 50 rpm throughout the session, 18 min, cycling (supervised, lab-based)	affect (↓)	0%
Decker (2016)	United States	–	30/30/0	39.3 ± 11.2	Inactive, Obese	Acute, within- subject (cross-over)	Traditional (single- component)	1 bout, work intervals: 4 × 3 min (115% of Watts at the ventilatory threshold), rest intervals: 4 × 2 min (85% of Watts at the ventilatory threshold), 20 min, cycling (supervised, lab-based)	affect (↓), enjoyment (↓)	20%
Ekkekakis (2010)	United States	–	27/27/0	42.5 ± 5.6	Inactive, Overweight/ Obese	Acute, within- subject (cross-over)	Traditional (single- component)	1 bout, incremental treadmill test began at a speed of 2.5 mph (1.11 m/s) and 0% grade for 2 min, walking (supervised, lab-based)	affect (↓)	20%
Freese (2014)	United States	6	47/47/0	52.1 ± 9.0	Inactive, Overweight/ Obese	Chronic, RCT	Traditional (single- component)	3 d/wk., work intervals: 4–8 × 30 s cycle (all-out sprints), rest intervals: 4–8 × 4 min (passive), 18–36 min, cycling (supervised, lab-based)	quality of life (↑)	21%
Heinrich (2014)	United States	8	23/13/10	26.8 ± 5.9	Inactive, Obese	Chronic, between-subject	Hybrid (multi- component)	3 d/wk., self-selected high-intensity, 30 min, aerobic (e.g., rowing), body-weight (9 fundamental movements), and weightlifting exercises in singular or multiple combinations (supervised, lab-based)	adherence (↑), enjoyment (↑)	25%
Kong (2016)	China	5	31/31/0	25.7 ± 2.4	Sedentary, Overweight/ Obese	Chronic, between-subject	Traditional (single- component)	4 d/wk., work intervals: 60 × 8 s (sprint), rest intervals: 60 × 12 s (passive), 20 min, cycling (supervised, lab-based)	enjoyment (↑)	13%
Little (2014)	Canada	–	10/8/2	40.6 ± 10.7	Inactive, Overweight/ Obese	Acute, within- subject (cross-over)	Traditional (single- component)	1 bout, work intervals: 10 × 1 min (~90% HRpeak), rest intervals: 10 × 1 min (passive), 20 min, cycling (supervised, lab-based)	affect (↔), enjoyment (↔)	0%
Martinez (2015)	United States	–	20/9/11	22.0 ± 4.0	Inactive, Overweight/ Obese	Acute, within- subject (cross-over)	Traditional (single- component)	1 bout, work intervals: 30, 60, and 90 s, rest intervals: 30, 60, and 90 s (passive), 24 min, cycling (supervised, lab-based)	affect (↓), enjoyment (↑)	0%
Ouerghi (2016)	Tunisia	8	12/0/12	18.2 ± 1.0	Inactive, Overweight/ Obese	Chronic, comparative study	Traditional (single- component)	3 d/wk., work intervals: 30 s (100–110% MAV), rest intervals: 30 s (active: 50% MAV), running (supervised, lab-based)	mood (including anxiety (↓) and depression (↓))	0%
Poon (2020)	Canada	8	24/0/24	48.1 ± 5.2	Inactive, Overweight/ Obese	Chronic, between-subject	Traditional (single- component)	3 d/wk., work intervals: 6–10 × 1 min (80–90% HRmax), rest intervals: 1 min (walk, 50% HRmax), 21–29 min, running (semi-supervised, semi-field)	enjoyment (↔)	0%
Ram (2021)	Australia	6	28/0/28	28.3 ± 6.9	Sedentary, Overweight/ Obese	Chronic, between-subject	Traditional (single- component)	3 d/wk., work intervals: 10 × 1 min (90–100% Wpeak); rest intervals: 9 × 1 min (active: 15% Wpeak) 19 min, cycling (supervised, lab-based)	affect (↑), enjoyment (↔)	16%
Reljic (2020)	Germany	12	65/36/29	48.7 ± 9.9	Sedentary, Obese	Chronic, RCT	Traditional (single- component)	2 d/wk., work intervals: 5 × 1 min (80–95% HRmax), rest intervals: 4 × 1 min (active), 9 min, cycling (supervised, lab-based)	quality of life (↑)	17%
Roy (2018)	New Zealand	48	104/59/45	43.5 ± 10.2	Sedentary, Overweight/ Obese	Chronic, within- subject	Traditional (single- component)	3 d/wk., work intervals: 3 × 30 s (maximal effort), 5–10 × 1 min (≥80% HRmax, RPE: 8), or 1 × 4 min (at the highest intensity that could be maintained), rest intervals: 1–3 min (active), 21–24 min, home-based exercises, sprinting, hill-walking, cycling, and exercise machines (unsupervised, field-based)	adherence (↓), enjoyment (↑)	20%
Santos (2021)	Canada	2	99/70/29	51.9 ± 9.6	Sedentary, Overweight/ Obese	Chronic, between-subject	Traditional (single- component)	5 d/wk., work intervals: 4–10 × 1 min (~77–95% HRmax), rest intervals: 3–9 × 1 min (~60% HRmax), 7–19 min, cycling, walking, or elliptical machine (semi-supervised, semi-field)	affect (↔), enjoyment (↔)	9%
Shepherd (2015)	United Kingdom	10	90/60/30	42.0 ± 11.0	Inactive, Overweight	Chronic, between-subject	Traditional (single- component)	3 d/wk., work intervals: 15–60 s (>90% HRmax), rest intervals: 45–120 s (active), 18–25 min, cycling (supervised, field-based)	affect (↑), vitality (↑)	9%
Sim (2014)	Australia	–	17/0/17	30.0 ± 8.0	Inactive, Overweight	Acute, within- subject (cross-over)	Traditional (single- component)	1 bout, work intervals: 60 s (100% VO_2_peak) or 15 s (170% VO_2_peak), rest intervals: 240 s (50% VO_2_peak) or 60 s (32% VO_2_peak), 30 min, cycling (supervised, lab-based)	enjoyment (↑)	0%
Smith-Ryan (2015)	United States	3	42/22/20	35.9 ± 12.1	Inactive, Overweight/ Obese	Chronic, between-subject	Traditional (single- component)	3 d/wk., work intervals: 10 × 1 min (90% PPO) or 5 × 2 min (80–100% PPO), rest intervals: 9 × 1 min or 4 × 1 min (passive), 15–20 min, cycling (supervised, lab-based)	enjoyment (↑)	0%
Sperlich (2017)	Germany	9	22/22/0	23.0 ± 2.0	Inactive, Overweight	Chronic, between-subject	Hybrid (multi- component)	3 d/wk., work intervals: 5–7 × 30–60 s, rest intervals: 30–60 s (passive), 3–6 rounds, 23–41 min, multi-stimulating, circuit-like, multiple-joint training (supervised, lab-based)	quality of life (↑)	0%
Vella (2017)	United States	8	17/10/7	26.2 ± 7.8	Sedentary, Overweight/ Obese	Chronic, between-subject	Traditional (single- component)	4 d/wk., work intervals: rest intervals: 10 × 1 min (75–80% HRR), rest intervals: 10 × 1 min (35–40% HRR), 20 min, running, cycling, or elliptical machine (semi-supervised, semi-field)	adherence (↔), enjoyment (↔)	11%

BMI, body mass index; HIIT, high-intensity interval training; HRmax, maximum heart rate; HRpeak, peak heart rate; HRR, heart rate reserve; MAV, maximal aerobic velocity; PPO, peak power output; PWR, peak work rate; RCT, randomized controlled trial; VO_2_peak, peak oxygen uptake; Wmax, maximal wattage; Wpeak, peak workload. ^1^ Sample size refers to participants who completed (not being recruited) the study; ^2^ Session duration (excluding warm-up and cool-down); ^3^ Dropout rate refers to overweight/obese participants who did not complete the HIIT intervention. ↑ indicates higher; ↓ indicates lower; ↔ indicates unchanged.

## Data Availability

Not applicable.

## Supplementary Materials

The following supporting information can be downloaded at: https://www.mdpi.com/article/10.3390/sports10050064/s1, Table S1: Search algorithms and results.
