# Supplementary material for: Psychological Adaptations to High-Intensity Interval Training in Overweight and Obese Adults: A Topical Review
Source: Sports (Basel). 2022 Apr 22;10(5):64. doi: 10.3390/sports10050064 (PMC9148041; doi:10.3390/sports10050064)
Supplement: Supplementary file 1 [file sports-10-00064-s001.zip › sports-1610497-supplementary.pdf]

Table S1. PubMed/MEDLINE search algorithms and results.

Search	Query	Items found
28	Search (4 AND 13 AND 27)	108
27	Search (14 OR 15 OR 16 OR 17 OR 18 OR 19 OR 20 OR 21 OR 22 OR 23 OR 24 OR 25 OR 26)	1,214,725
26	Search mental health[Title/Abstract]	162,123
25	Search perceptual[Title/Abstract]	52,822
24	Search psychological[Title/Abstract]	257,887
23	Search arousal[Title/Abstract]	30,938
22	Search feeling[Title/Abstract]	36,409
21	Search depression[Title/Abstract]	384,167
20	Search anxiety[Title/Abstract]	228,470
19	Search mood[Title/Abstract]	83,450
18	Search quality of life[Title/Abstract]	319,872
17	Search affect[Title/Abstract]	745,717
16	Search enjoyment[Title/Abstract]	6,413
15	Search compliance[Title/Abstract]	129,827
14	Search adherence[Title/Abstract]	139,852
13	Search (5 OR 6 OR 7 OR 8 OR 9 OR 10 OR 11 OR 12)	4,619
12	Search HIIE[Title/Abstract]	284
11	Search HIIT[Title/Abstract]	1,599
10	Search high-intensity intermittent exercise[Title/Abstract]	274
9	Search high-intensity intermittent training[Title/Abstract]	83
8	Search high-intensity interval exercise[Title/Abstract]	468
7	Search high-intensity interval training[Title/Abstract]	2,373
6	Search interval exercise[Title/Abstract]	861
5	Search interval training[Title/Abstract	3,632
4	Search (1 OR 2 OR 3)	364,265
3	Search overweight[Title/Abstract]	79,678
2	Search obese[Title/Abstract]	139,477
1	Search obesity[Title/Abstract]	287,875
